# Syndrome de Lyell staphylococcique: à propos d’un cas

**DOI:** 10.11604/pamj.2021.39.177.22171

**Published:** 2021-07-06

**Authors:** Said Khallikane, Mohamed Moutaoukil, Hanane Delsa

**Affiliations:** 1 Service de Réanimation Polyvalente, Troisième Hôpital Militaire, Laayoune, Royaume du Maroc,; 2 Service de Réanimation Polyvalente, Quatrième Hôpital Militaire, Dakhla, Royaume du Maroc,; 3 Service de Gastro-Entérologie, Université Mohammed VI des Sciences de la Santé (UM6SS), Hôpital Cheikh Khalifa Bin Zayd Al Nahyan, Casablanca, Royaume du Maroc

**Keywords:** Syndrome de Lyell, nécrolyse toxique épidermique, syndrome de Lyell staphylococcique, exotoxines, à propos d’un cas, Lyell syndrom, toxic epidermal necrolysis, staphylococcal scalded skin syndrom, staphylococcus, exotoxins, case report

## Abstract

L´épidermose staphylococcique aiguë généralisée est une dermatose bulleuse médiée par des endotoxines staphylococciques exfoliatrices. L´affection touche le plus souvent les jeunes enfants. Nous rapportons le cas d´un nourrisson de 6 mois, ayant présenté une angine dans les jours précédant l´érythrodermie bulleuse, et dont la biopsie cutanée a montré l’aspect caractéristique de l´épidermolyse staphylococcique. L´évolution était rapidement défavorable et le nourrisson est décédé dans un tableau de choc septique réfractaire. Le terme de syndrome de la peau ébouillantée (SSSS), a été séparé de la nécrolyse épidermique d´origine toxique ou allergique par Lyell en l´aspect anatomique opposé de ces deux entités: dans le syndrome de la peau ébouillantée, le décollement cutané se fait par clivage de la partie superficielle de l´épiderme au niveau de la couche granuleuse, alors que dans le syndrome de Lyell toxique, le clivage siège plus profondément au niveau du corps muqueux.

## Abstract (en)

Staphylococcal scalded skin syndrom is a bullous dermatosis induced by exfoliating staphylococcal exotoxins. Children are most often affected. We report the case of a 6-month-old infant who had angina in the few days before leading up to bullous erythroderma and whose skin biopsy showed characteristic appearance of staphylococcal scalded skin syndrom. The development was rapidly unfavourable and the infant died in a refractory septic shock chart, despite the introduction of norepinephrine and anti-SAMR antibiotic therapy. The term staphylococcal scalded skin syndrome (SSSS) was separated from the toxic or allergic epidermal necrolysis by Lyell into the opposite anatomical aspect of these two entities: in scalded skin syndrome, Skin detachment is done by cleavage of the superficial part of the epidermis at the granular layer, while in toxic Lyell syndrome, the cleavage sits deeper at the level of the mucous body.

## Introduction

L´épidermolyse staphylococcique aiguë est parfois appelée syndrome de 4S d´après la dénomination anglaise (staphylococcal scalded skin syndrome). Elle affecte préférentiellement les nouveau-nés et les jeunes enfants mais, dans de rares cas, elle touche également les adultes. Actuellement, l´incidence de cette pathologie est en augmentation dans tous les groupes d´âge. La résistance aux traitements antibiotiques conventionnels devient aussi une réalité émergente.

## Patient et observation

Nous rapportons le cas d´un nourrisson de 6 mois, sans antécédents pathologiques notables, fût hospitalisé en réanimation polyvalente A de l´HER pour une érythrodermie, associée à une déshydratation aiguë sévère. A son admission, il présentait un érythème généralisé prédominant dans les grands plis et en péribuccal qui était associé à des décollements bulleux flasques et squameux avec une hypotension 60/30, tachycardie 180 bpm sans fièvre, le signe de Nikolski était présent. Sur le plan anamnestique, le patient avait présenté la semaine précédente une rhinopharyngite avec une angine traitées par amoxicilline et paracétamol. Après une réanimation hydroélectrolytique, antibiothérapie à base d´oxacilline et un parage des lésions par une crème émolliente, la stabilité hémodynamique était obtenue. Au cours de l´hospitalisation la nécrolyse épidermique s´est rapidement étendue laissant apparaitre de larges surfaces érythémateuses érodées et suintantes recouvertes de lambeaux épidermiques les muqueuses restaient intactes un syndrome biologique inflammatoire ainsi qu´une légère insuffisance rénale fonctionnelle. Trois diagnostics différentiels furent évoqués devant ce tableau clinique: une nécrolyse épidermique toxique d´origine médicamenteuse (syndrome de Lyell) [1], une épidermolyse staphylococcique aiguë et un choc staphylococcique. La biopsie cutanée met en évidence un clivage intra-épidermique superficiel siégeant au niveau de la couche granuleuse, ainsi qu´un œdème et un infiltrat lymphoïde dans le derme superficiel. Cet aspect était caractéristique de l´épidermolyse staphylococcique aiguë. Les prélèvements urinaires, cutanés étaient négatifs sur le plan bactériologique. Un staphylocoque doré coagulase positif méticilline sensible fût mis en évidence par hémoculture. L´évolution était rapidement défavorable et le nourrisson est décédé dans un tableau de choc septique réfractaire, malgré l´introduction de la noradrénaline et la mise sous antibiothérapie anti-SAMR.

## Discussion

Epidermolyse staphylococcique aiguë est la destruction superficielle mais généralisée de l´épiderme causée par une toxine exfoliatrice épidermolytique produite par certaines souches de Staphylocoque aureus parfois méticilline résistantes [2]. Pendant de nombreuses années, ce syndrome et la nécrolyse épidermique toxique furent confondus en une seule entité sous l´éponyme de syndrome de Lyell [3]. Ces deux affections aujourd´hui bien séparées sur le plan nosologique se distinguent nettement sur le plan histologique. L´épidermolyse staphylococcique aiguë est caractérisée par un clivage intra-épidermique au niveau de la couche granuleuse sans atteinte de l´épiderme sous-jacent alors que la nécrolyse épidermique toxique iatrogène est caractérisée par un décollement de tout l´épiderme par nécrose apoptotique généralisée des kératinocytes. Le diagnostic de l´épidermolyse staphylococcique aiguë généralisée repose finalement sur la combinaison d´une érythrodermie desquamative avec formation de bulles, et tout accompagné de la démonstration histologique d´un clivage intra -épidermique au niveau de la couche granuleuse [3,4]. L´affection atteint surtout les enfants et les nouveau-nés les adultes sont rarement touchés, probablement en raison d´une meilleure métabolisation et excrétion des exotoxines staphylococciques. Généralement ce syndrome survient dans les jours suivant le début d´une infection focale muqueuse (rhinopharyngite, otite, conjonctivite), d´une infection cutanée (impétigo) ou plus exceptionnellement d´un abcès sous-cutané, voire même d´une endocardite [5].

L´entrée du staphylocoque dans l´organisme se fait souvent à partir d´un foyer nasal asymptomatique qui est retrouvé dans 35% de la population. Chez l´adulte, deux facteurs de risque majeur ont été identifiés: d´une part tout état d´immunodéficience (cancer, infections particulières et médications immunosuppressives) et d´autre part, l´insuffisance rénale [3,4,6,7]. Cette dernière engendre une diminution de la clairance des toxines. Toutefois la fonction rénale souvent altérée dans cette pathologie ne peut tout expliquer. L´immunosuppression réduit la production d´anticorps antitoxines staphylococciques qui contribuent à limiter les dégâts au point d´entrée de l´agent pathogène [4]. Quelques facteurs de moindre risque sont également à prendre en considération tels que la macération chez des plis chez les patients obèses ou diabétiques, les dermatoses chroniques (dermatite atopique, eczéma, psoriasis) et les brulures. La pathogénie de l´épidermolyse staphylococcique aiguë reste mal connue, deux types de toxines appelés ETA ou ETB sont produites par les souches de staphylocoques responsables [4]. La production d´ETA est génétiquement encodée dans la bactérie tandis que celle d´ETB est médié par un plasmide. La toxine la plus fréquemment excrétée et le plus souvent incriminé est ETA. Dans de rares cas on observe l´action conjointe de l´ETA et ETB. Cliniquement, il n´est pas possible de distinguer l´effet de l´une ou de l´autre de ces toxines exfoliatrices. L´exotoxine circulante se lierait à la desmogléine de type I, une protéine importante dans la structure et la fonction des desmosomes, unissant les kératinocytes de la couche granuleuse de l´épiderme. Le mécanisme aboutissant à l´acantholyse n´est pas encore connu. Il pourrait résulter d´une similitude structurelle de l´exotoxine avec des sérines protéases [8].

La frontière nosologique entre l´épidermolyse staphylococcique aiguë et certaines formes graves du syndrome de toxique staphylococcique est parfois incertaine. En effet, diverses souches de staphylocoques sont capables de sécréter conjointement les toxines exfoliantes et érythrogènes TSST-1 responsables de ces affections [8]. Sur le plan clinique, le choc toxique staphylococcique est caractérisé initialement par un érythème généralisé, mais plus intense sur le tronc. Il peut ressembler à l´exanthème de la scarlatine. Habituellement l´éruption n´est ni douloureuse, ni prurigineuse et elle forme rarement des bulles. Il s´y associe fréquemment une hyperhémie conjonctivale, pharyngée et vaginale. Une desquamation qui touche en particulier les paumes et les doigts et les plantes survient 1 à 2 semaines après le début de l´éruption [2-4].

Traitement de l´épidermolyse staphylococcique aiguë doit idéalement se faire en unité de soins intensifs ou en réanimation car les traitements utilisés, l´évolution et les complications de la maladie se rapprochent de celles des brulés graves [3,7]. Une antibiothérapie parentérale anti-staphylococcique adaptée à l´antibiogramme est nécessaire. En revanche les antibiotiques topiques sont souvent inefficaces. Les mesures habituelles d´antisepsie de contrôle des pertes liquidiennes avec réhydratation et de contrôle de la température corporelle s´imposent également. L´usage d´un lit fluidisé est utile. Alors que l´évolution est souvent favorable chez l´enfant, taux de mortalité chez l´adulte atteint 50%. Le décès survient à la suite d´une septicémie ou par décompensation d´une pathologie préalable [2-4].

## Conclusion

Le terme de syndrome de la peau ébouillantée (SSSS), a été séparé de la nécrolyse épidermique d´origine toxique ou allergique par Lyell en l´aspect anatomique opposé de ces deux entités: dans le syndrome de la peau ébouillantée, le décollement cutané se fait par clivage de la partie superficielle de l´épiderme au niveau de la couche granuleuse, alors que dans le syndrome de Lyell toxique, le clivage siège plus profondément au niveau du corps muqueux. Les complications sont rares et la mortalité très faible. Cependant, il existe des formes étendues, en particulier sur varicelle, qui peuvent s´accompagner de déperdition liquidienne, d´hypothermie, d´un syndrome de fuite capillaire et de choc. Le syndrome de Ritter représente la même affection chez le nouveau-né.
